# Silicon Oxide Etching Process of NF_3_ and F_3_NO Plasmas with a Residual Gas Analyzer

**DOI:** 10.3390/ma14113026

**Published:** 2021-06-02

**Authors:** Woo-Jae Kim, In-Young Bang, Ji-Hwan Kim, Yeon-Soo Park, Hee-Tae Kwon, Gi-Won Shin, Min-Ho Kang, Youngjun Cho, Byung-Hyang Kwon, Jung-Hun Kwak, Gi-Chung Kwon

**Affiliations:** 1 Department of Electrical and Biological Physics, Kwangwoon University, 20 Kwangwoon-ro, Nowon-gu, Seoul 01897, Korea; dnwo424@naver.com (W.-J.K.); bebe403@naver.com (I.-Y.B.); oneaeo@daum.net (J.-H.K.); small2008@naver.com (Y.-S.P.); HeeTae_Kwon@outlook.com (H.-T.K.); swat2100@naver.com (G.-W.S.); 2 Department of Nano-Process, National Nanofab Center (NNFC), 291 Daehak-ro, Yuseong-gu, Daejeon 34141, Korea; kmh@nnfc.re.kr; 3 SK Materials Co., Ltd., 110-5, Myeonghaksandan-ro, Yeondong-myeon, Sejong 30068, Korea; choyongjun@sk.com (Y.C.); qudgid@sk.com (B.-H.K.); jhkwak@sk.com (J.-H.K.)

**Keywords:** nitrogen oxide trifluoride, nitrogen fluoride oxide, reactive ion etch, silicon oxide etch

## Abstract

The use of NF_3_ is significantly increasing every year. However, NF_3_ is a greenhouse gas with a very high global warming potential. Therefore, the development of a material to replace NF_3_ is required. F_3_NO is considered a potential replacement to NF_3_. In this study, the characteristics and cleaning performance of the F_3_NO plasma to replace the greenhouse gas NF_3_ were examined. Etching of SiO_2_ thin films was performed, the DC offset of the plasma of both gases (i.e., NF_3_ and F_3_NO) was analyzed, and a residual gas analysis was performed. Based on the analysis results, the characteristics of the F_3_NO plasma were studied, and the SiO_2_ etch rates of the NF_3_ and F_3_NO plasmas were compared. The results show that the etch rates of the two gases have a difference of 95% on average, and therefore, the cleaning performance of the F_3_NO plasma was demonstrated, and the potential benefit of replacing NF_3_ with F_3_NO was confirmed.

## 1. Introduction

Since the 1980s, the use of NF_3_ plasmas in semiconductor, display, and solar cell processing applications has been investigated [1]. NF_3_ plasma is used to etch various thin films under reactive ion etching (RIE) conditions [2,3] or to clean a plasma-enhanced chemical vapor deposition (PECVD) chamber [4,5]. Cleaning the PECVD chamber is performed by supplying ions and radicals for cleaning through a remote plasma source (RPS) [6] or by directly supplying ions and radicals through an in situ plasma discharge [7]. In addition, NF_3_ is attracting attention as a new etching technology, such as cryogenic electron beam induced etching (EBIE) [8,9] and highly selective etching [10]. NF_3_ has a high etch rate, etching efficiency, and a relatively high chemical stability [2,11]. Accordingly, the use of NF_3_ is significantly increasing every year. However, NF_3_ is a greenhouse gas with a very high global warming potential of 16,100 and a lifespan of 500 years [12]. The contribution of NF_3_ to the radiative forcing in the Earth’s atmosphere is very small, ~0.01%, in 2011, but the use of NF_3_ is increasing every day; therefore, this number continues to increase [13]. The share of NF_3_ in fluorinated gases increased from 13–28% in 2005 to 17–36% in 2010, and NF_3_ is currently the most widely used and released fluorinated gas [1,14]. Thus, it was included in the second commitment period of the Kyoto Protocol as the seventh greenhouse gas whose emissions are to be regulated [15].

Studies have been conducted to replace NF_3_ [16,17]. Among them, F_3_NO was considered a candidate gas to replace NF_3_ [18]. Similar to NF_3_, F_3_NO does not contain perfluorocarbons. In addition, because its molecule has an N=O bond, its atmospheric life is relatively short, and thus, its contribution to global warming is expected to be less than that of NF_3_. The etch rate of F_3_NO is almost the same as that of NF_3_. However, further studies on F_3_NO have not been conducted, and information on the mechanism occurring in the F_3_NO plasma during the cleaning process is insufficient. Therefore, research on the F_3_NO plasma to replace NF_3_ is urgently needed.

The present study focused on evaluating and analyzing the properties of F_3_NO plasma. Accordingly, the cleaning abilities of NF_3_ and F_3_NO were compared, and the characteristics of the F_3_NO plasma were analyzed. The cleaning ability of the F_3_NO plasma was evaluated by etching a SiO_2_ film previously deposited on a Si wafer sample. Although the etching time of the small sample and the cleaning time of the PECVD chamber may not coincide, it was considered suitable for the evaluation of the cleaning ability of the gas. To analyze the etch mechanism, the etching plasma was diagnosed using a residual gas analyzer (RGA) and a high-voltage probe. The reactions in the F_3_NO plasma were predicted by comparing the types and intensity of ions generated in the NF_3_ and F_3_NO plasmas. In addition, the etch rates of the SiO_2_ thin film using the NF_3_ and F_3_NO plasmas were compared to confirm whether the F_3_NO plasma can replace NF_3_.

## 2. Materials and Methods

**Figure 1:**
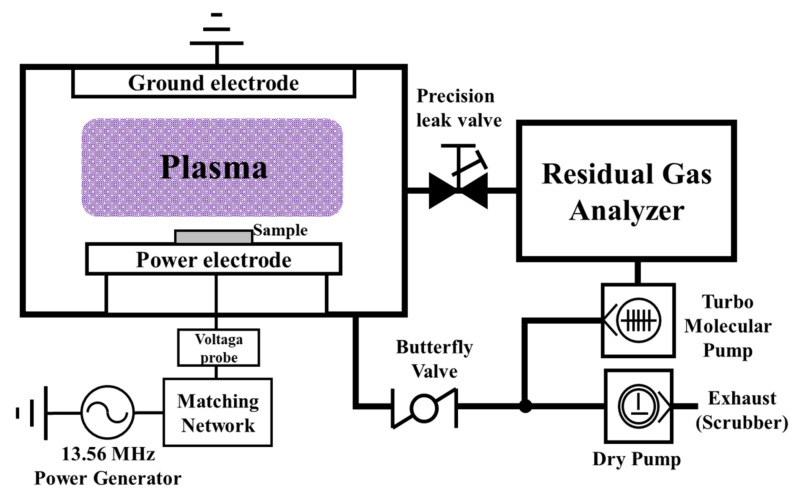
Plasma etching equipment setup.

The plasma etching equipment used in this study is shown in Figure 1. We manufactured the RIE equipment with a direct capacitively coupled plasma chamber to measure the plasma etching characteristics. The detailed geometry of the device has been shown in a previous study [19]. A coolant path was formed in the electrode, which was designed to maintain a constant temperature (15 °C) during the process.

The sample used for etching was a SiO_2_ thin film deposited on a Si wafer. The size of the sample was 30 mm × 30 mm, and the thickness of the thin film was 2 μm. NF_3_ and F_3_NO were used as process gases for etching. The F_3_NO used in the experiments was manufactured and supplied by SK Materials (Sejong, Korea) and the National NanoFab Center (Daejeon, Korea). The purity of the provided F_3_NO gas was 99.995%. The injected process gas was controlled using mass flow controllers. The process flow rate was fixed at 120 sccm. After the injection of the process gas, the process pressure was adjusted using a butterfly valve. The working pressure range was 130–270 mTorr. This working pressure range was set to confirm the possibility of cleaning in situ with a small amount of gas in the chamber while reducing the amount of cleaning gas without any additional high flow MFC configuration. After reaching the process pressure, the discharge power was applied through the RF power generator. The RF input power range was 240–400 W, and the process time was fixed at 3 min.

Mass spectrometry measurements were performed using an RGA (RGA 300, SRS, Sunnyvale, CA, USA). As shown in Figure 1, the RGA is equipped with a differential pump system. The pressure of the differential pump system was fixed at 0.8 mTorr regardless of the process pressure. At this time, the ionization energy of the RGA was fixed at 70 eV. The DC self-bias (DC offset) was measured using a high-voltage probe to determine the characteristics of the NF_3_ and F_3_NO plasmas. The etching rate of the thin film was evaluated after etching by measuring its thickness using the spectroscopic reflectometry method via the S-TRC series (Wonwoo Systems Co., Ltd., Seoul, Korea) [20].

## 3. Results and Discussion

**Figure 2:**
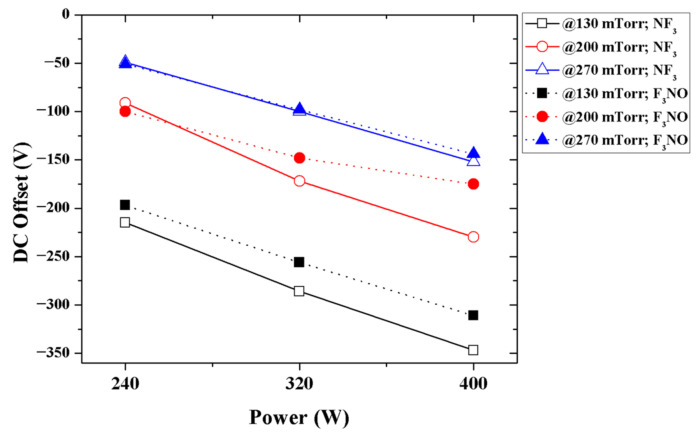
DC offset of the RF electrode during plasma discharge at a total flow rate of 120 sccm.

To compare the characteristics of the NF_3_ and F_3_NO plasmas and to understand the characteristics of the latter, a DC offset measurement during the RIE plasma discharge was performed (Figure 2). The F_3_NO plasma showed a similar DC offset value very close to that of the NF_3_ plasma.

The NF_3_ plasma has a very high electronegativity [21,22]. Therefore, when compared with fluorocarbon plasmas, the NF_3_ plasma shows a relatively low DC offset value. Plasma with a high electronegativity tends to be unstable and easily collapses. In this case, the NF_3_ plasma is discharged only in a part of the chamber, and the plasma collapses in other parts; therefore, these parts may not be exposed to the plasma. Alternatively, a constant discharge may not occur but may flicker and cause discharge.

The comparison results of the DC offset of the NF_3_ and F_3_NO plasmas show that the electronegativity of the F_3_NO plasma can also be quite considerable. In the case of the NF_3_ plasma, an inert gas (e.g., He, Ar) was diluted and discharged to increase the stability and uniformity of the plasma and enhance etch rates [23,24,25,26]. Inert gases rarely participate in chemical reactions in the plasma but can have a great influence on the discharge process. The DC offset result indicates that the characteristics of the F_3_NO plasma can also be comparable to those of the NF_3_ plasma. Thus, in future studies, dilution with an inert gas, such as Ar, is recommended.

**Figure 3:**
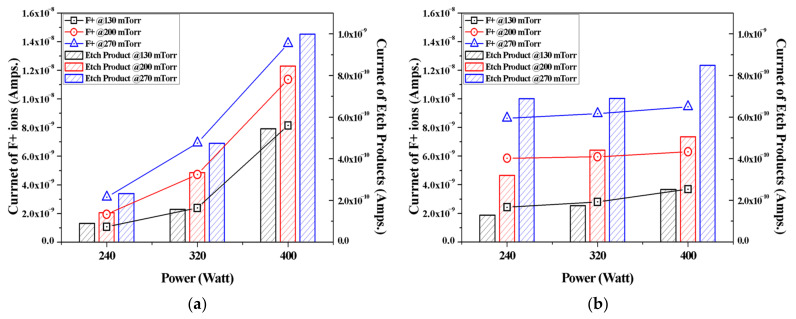
(**a**) Intensity of F+ ions and the sum of the etch product ions (SiF+, SiF_2_+, and SiF_3_+) during NF_3_ plasma silicon oxide etching as functions of the input power for various working pressures; (**b**) Intensity of F+ ions and the sum of the etch product ions (SiF+, SiF_2_+, and SiF_3_+) during F_3_NO plasma silicon oxide etching as functions of the input power for various working pressures.

Figure 3 shows the current of F+ ions and etch products (SiF, SiF_2_, and SiF_3_) generated from the NF_3_ and F_3_NO plasmas when etching SiO_2_ thin films. As the pressure and power increased, the intensity of the peak of F ions and the etch product also increased. At this time, when the pressure or discharge power was low, more F ions were generated in the F_3_NO plasma than in the NF_3_ plasma. In addition, the lower the discharge power, the higher the generation of etch products in F_3_NO than in NF_3_. These findings confirmed that the etching of F_3_NO occurs more actively at low power and pressure. In addition, as the power increases, the intensity of F ions in NF_3_ rapidly increases, whereas in the F_3_NO plasma, the F ions gradually increase even if the discharge power of F increases. As will be shown further, this trend is similar for other ions as well. Consequently, the plasma density of F_3_NO reacts more slowly to the change of the discharge power compared to NF_3_.

**Figure 4:**
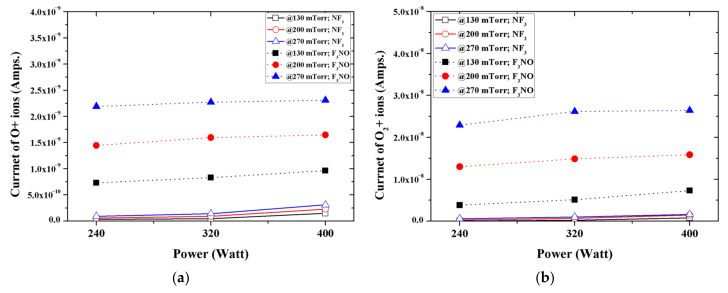
Intensity of: (**a**) O+ ions; (**b**) O_2_+ during plasma silicon oxide etching as functions of input power for various working pressures.

Among the ions generated in the NF_3_ and F_3_NO plasmas during the silicon oxide thin film etching, O and O_2_ were more common in the F_3_NO plasma under all conditions (Figure 4). Compared with the F_3_NO plasma, O and O_2_ ions hardly occurred in the NF_3_ plasma. In the case of the F_3_NO plasma, O and O_2_ ions were simultaneously generated by O contained in F_3_NO and O_2_ gas already present in the chamber, whereas the NF_3_ plasma generated O and O_2_ ions only from O_2_ gas existing in the base. Therefore, when the pressure increased, the intensity of O and O_2_ ions generated in the NF_3_ plasma was almost unchanged, whereas in the F_3_NO plasma, when the pressure increased, O and O_2_ ions appeared to rapidly increase compared to the NF_3_ plasma.

**Figure 5:**
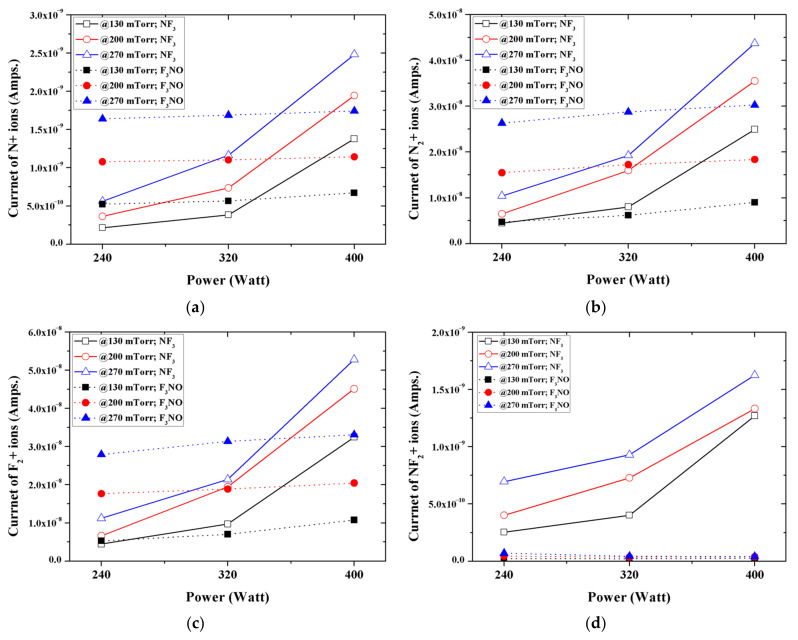
Intensity of: (**a**) N+ ions; (**b**) N_2_+ ions; (**c**) F_2_+ ions; (**d**) NF_2_+ ions during plasma silicon oxide etching as functions of the input power for various working pressures.

Figure 5 shows the peak intensities of the major ions generated in the NF_3_ and F_3_NO plasmas during the etching of a silicon oxide thin film. N, N_2_, and F_2_ ions occurred more in F_3_NO at low discharging powers and more in NF_3_ at higher discharging powers. Conversely, NF_2_ ions occurred more in the NF_3_ plasma under all conditions.

**Figure 6:**
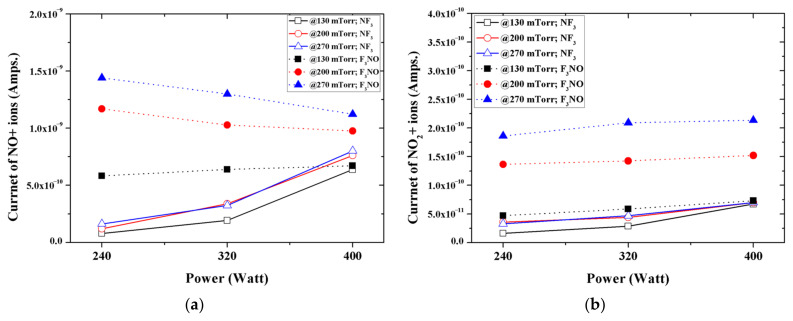
Intensity: of (**a**) NO+ ions; (**b**) NO_2_+ ions during plasma silicon oxide etching as functions of the input power for various working pressures.

Figure 6 shows the intensity of NO and NO_2_ ions generated in the NF_3_ and F_3_NO plasmas during the etching of silicon oxide thin films. When the discharge power was low, the NO ions generated in the F_3_NO plasma were more than those of the NF_3_ plasma (numerical value). However, as the discharge power increased, the number of NO ions in the F_3_NO plasma gradually decreased, whereas those in the NF_3_ plasma rapidly increased and became almost the same as the peak intensity of NO ions generated in the F_3_NO plasma. Conversely, many more NO_2_ ions occurred in the F_3_NO plasma under all conditions.

(1) $$\mathrm{In}\;\mathrm{the}\;{\mathrm{NF}}_{3}\;\mathrm{and}\;F_{3}\mathrm{NO}\;\mathrm{plasmas},\;\mathrm{NO}\;\mathrm{was}\;\mathrm{produced}\;\mathrm{through}\;\mathrm{the}\;\mathrm{following}\;\mathrm{reaction}:N_{2}\;(A^{3}\Sigma_{u}^{+})\;O\;\to \;\mathrm{NO}\;+\;N.$$

Metastable N_2_ is produced mostly by collisions with a high-energy electron, making the mechanism more significant in an electronegative gas discharge, such as NF_3_ and F_3_NO. At a higher discharge power, higher energy electrons are supplied, thus increasing the density of NO ions [27]:

N + O_2_ → NO + O. (2)

A reaction involving atomic nitrogen that resulted in a different density of NO ions in the F_3_NO plasma and the NF_3_ plasma was more important in an oxygen-rich plasma [28,29]. As shown in Figure 4a, because F_3_NO is an oxygen-rich plasma, reaction (2) became significant, and a large amount of NO ions were produced.

In the F_3_NO plasma, NF is decomposed through the following reaction.

NF_2_ + O → F + FNO. (3)

The species produced in the primary reactions led to secondary reactions which formed NO_2_ ions through the following exothermic reactions:

FNO + O → F + NO_2_, (4)

FO + NO → F + NO_2_, (5)

N_2_O + NO → N2 + NO_2_, (6)

NO + O_3_ → O_2_ + NO_2_. (7)

In the case of the NF_3_ plasma, the above reaction is not significant because the number of O ions is remarkably small, but F_3_NO causes a more significant reaction. Therefore, the number of NF_2_ and NF ions are fewer in the F_3_NO plasma than in the NF_3_ plasma, whereas ions such as N, N_2_, and F are present in similar amounts in the F_3_NO plasma and the NF_3_ plasma.

In the NF_3_ plasma, NO ions may be generated through the bonding of O and N ions, which are etching by-products, or through a process in which N ions form Si–O–N bonding on the surface of the silicon oxide thin film [30]. In the F_3_NO plasma, NO is formed by the N and O ions contained in F_3_NO. In the case of F_3_NO, this condition becomes the main mechanism. NO formed in this way forms NO_2_ through the following reaction:

NO + O + M → NO_2_ + M. (8)

In the NF_3_ plasma, because the number of O ions was remarkably small, this oxidation reaction occurred only to a small extent. Conversely, in the F_3_NO plasma, as the number of O ions was much larger, the extent of the oxidation reaction was more significant. When the discharge power was increased, the number of NO formed was almost unchanged, but the peak of NO ions decreased due to the considerable oxidation of NO.

**Figure 7:**
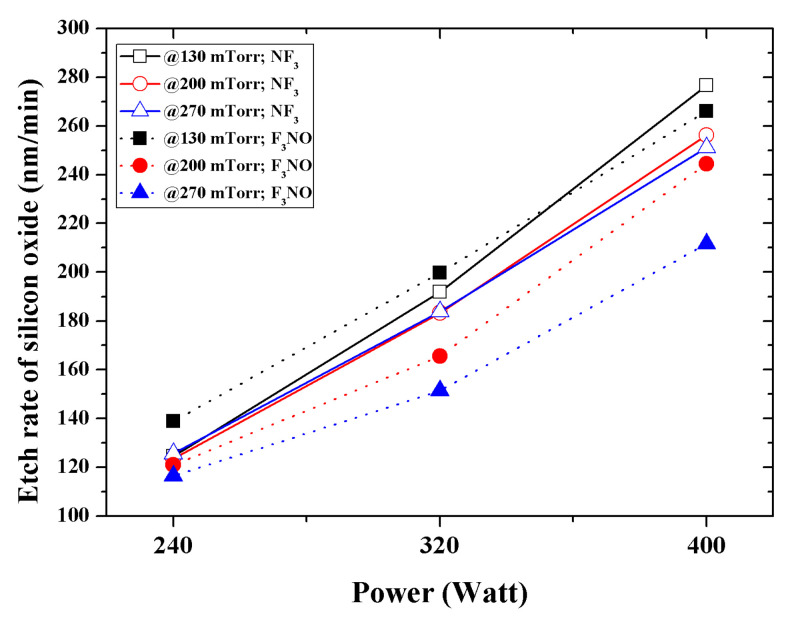
Etch rate as a function of input for various working pressures during plasma silicon oxide etching at a total flow rate of 120 sccm.

Figure 7 shows the SiO_2_ etch rate according to the discharge power and process pressure. The results imply that the lower the pressure and discharge power, the higher the etch rate of F_3_NO. This is because when the pressure and discharge power is low, chemical etching occurs more easily because the number of F ions generated is greater in F_3_NO. Conversely, the DC offset at a low power has a small absolute value for the NF_3_ and F_3_NO plasmas; therefore, the ion bombardment energy does not have a significant effect on etching. When the discharge power was increased, the DC offset value of the NF_3_ plasma became larger than that of the F_3_NO plasma, so the etch rate of NF_3_ also became higher. When the discharge pressure and discharge power increased, the intensity of F ions also increased as NF_3_ increased, so the etch rate decreased in F_3_NO. Furthermore, the etch rate of silicon oxide during F_3_NO plasma etching was approximately 95.0% of the rate during NF_3_ plasma etching.

**Figure 8:**
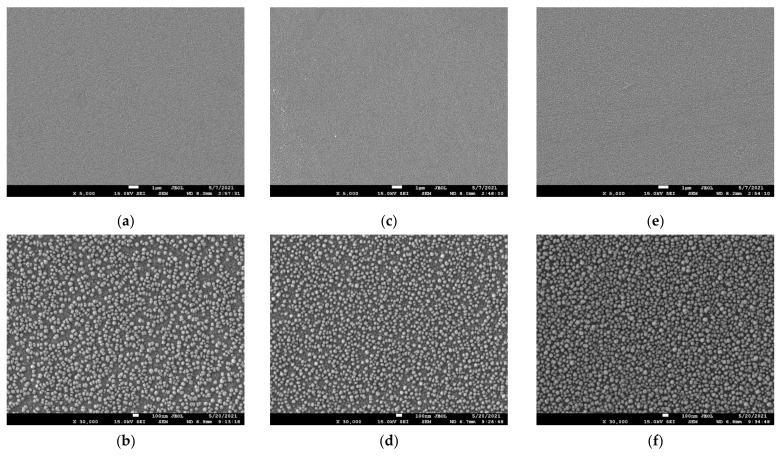
Surface of the SEM images of the SiO_2_ samples: without etching of (**a**) ×5000, (**b**) ×30,000, at 400 W discharge power and 270 mTorr process pressure with NF_3_ plasma of (**c**) ×5000, (**d**) ×30,000 and F_3_NO plasma of (**e**) ×5000 and (**f**) ×30,000.

We performed SEM measurements to determine whether O or N ions present in the plasma had a negative effect on the etching quality. Figure 8 shows a SiO_2_ surface of SEM images after the etching process at 400 W discharge power and 270 mTorr pressure. For accurate SEM measurements, a platinum coating was applied on the surface by sputtering. The round shape particles in the figures are platinum nanoparticles from the platinum coating. The size of these nanoparticles is in order of several nanometers. Besides platinum nanoparticles, no other structures such as cracks or holes were found on the surface. No significant difference was observed between the SEM images of the unprocessed and processed surface of SiO_2_. Therefore, it was confirmed that O or N ions in F_3_NO did not have a negative effect on the etching quality.

**Figure 9:**
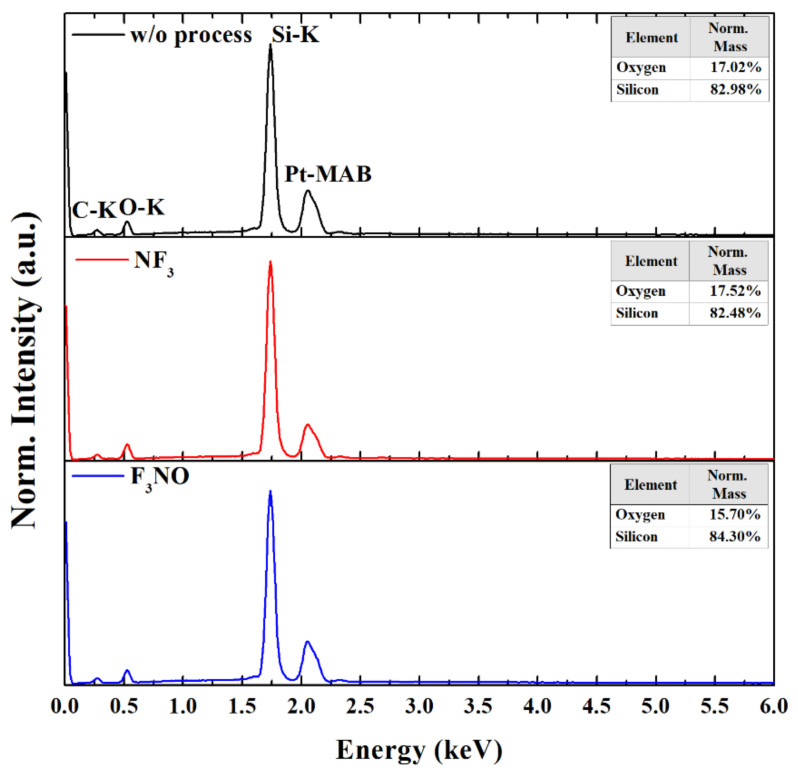
EDS spectra of the SiO_2_ surface without etching, at 400 W discharge power and 270 mTorr process pressure with NF_3_ plasma and F_3_NO plasma.

Figure 9 shows EDS spectra of the SiO_2_ surface without process and after etching at a 400 W discharge power and 270 mTorr pressure with the NF_3_ and F_3_NO plasmas. The C peak in the EDS spectra was caused either by carbon contamination or by the window in the detector. Except for carbon and platinum (from the platinum coating), no peaks other than O and Si were found in the EDS spectra. This indicates that nitrogen, oxygen, or NO did not chemically contaminate the SiO_2_ surface during the etching process.

The mass ratio of silicon and oxygen is noticeable in the EDS spectra. The mass ratio of silicon and oxygen on the SiO_2_ surface is almost the same when the process is not performed and when the NF_3_ etching process is performed. However, the mass ratio of O appears less on the SiO_2_ surface after F_3_NO etching. This may be caused by the following reaction on the SiO_2_ surface during F_3_NO etching.

O(s) + NO(g) → NO_2_(g). (9)

As above, NO ions absorb O in the SiO_2_ surface to decrease surface oxidation [31]. Therefore, the F_3_NO etched SiO_2_ surface has a smaller oxygen mass ratio than the NF_3_ etched SiO_2_ surface. Moreover, this de-oxidation process increases the Si etching rate, especially during etching with F_3_NO at low pressure and low power with a significant quantity of NO ions.

## 4. Conclusions

The DC offset was measured during NF_3_ and F_3_NO plasma discharges. Compared with the DC offset of the NF_3_ plasma, the F_3_NO plasma showed an almost similar DC offset value. This finding confirms that the F_3_NO plasma, similar to the NF_3_ plasma, can have a very high electronegativity. Moreover, the NF_3_ plasma, similar to the F_3_NO plasma, may also exhibit unstable or non-uniform characteristics. Therefore, the diluent of an inert gas into the F_3_NO plasma can be effective.

The ions generated in the NF_3_ plasma and the F_3_NO plasma during the etching of the SiO_2_ thin film were measured through the RGA. In the case of F ions, when the discharge power and discharge pressure were low, more F_3_NO plasmas were generated than NF_3_ plasmas. The result was the same for the etching by-products (SiF, SiF_2_, and SiF_3_). In addition, as the power increased, the intensity of F ions in NF_3_ rapidly increased, whereas in the F_3_NO plasma, the ions of F gradually increased even if the discharge power of F increased. This result showed a similar trend for other ions afterward, implying that the plasma density of F_3_NO reacts more slowly to the change of the discharge power compared to NF_3_. Ions O and O_2_ generated during the plasma discharge were much more significant in the F_3_NO plasma than in the NF_3_ plasma, which is attributed to the O ions contained in F_3_NO. Furthermore, the intensity of O ions can affect the etching mechanism of the F_3_NO plasma. F_2_, N, N_2_ ions occur more in F_3_NO at a low discharge power and occur more in NF_3_ at a high discharge power. In contrast, NF_2_ ions are much higher in NF_3_ ions under all conditions. O ions in F_3_NO cause a reaction to decompose NF_2_. When the discharge power is low, the NO ions of F_3_NO are generated in higher amounts compared to the NF_3_ plasma. However, as the discharge power increases, the number of NO ions in F_3_NO gradually decreases, whereas the NO ions in the NF_3_ plasma rapidly increase, and the intensity of the peak of NO generated in F_3_NO becomes almost similar. Conversely, NO_2_ ions occur more in the F_3_NO plasma under all conditions. In the F_3_NO plasma, many NO ions are generated in the process of decomposing NF_2_. However, when the pressure increases, NO ions are oxidized by O ions to form NO_2_ ions, and thus the number of NO ions decreases. Through this oxidation reaction, many more NO_2_ ions are generated in the F_3_NO plasma than in the NF_3_ plasma.

The SiO_2_ etch rates of the NF_3_ and F_3_NO plasmas were compared. The results show that the lower the pressure and discharge power, the higher the etch rate of F_3_NO. This is because the intensity of F ions is higher in the F_3_NO plasma at low pressure. As the discharge power increases, the intensity of F ions in the NF_3_ plasma increases. Therefore, the etch rate of the NF_3_ plasma increases. The etch rate of silicon oxide during F_3_NO plasma etching was approximately 95.0% of the NF_3_ plasma etching rate.

To compare the etch qualities, SEM measurements were performed. There was no difference between the unetched and etched SiO_2_ surface with the NF_3_ and F_3_NO plasmas. Therefore, we found that N or O ions in F_3_NO did not negatively affect the etch quality. The results of this study confirm the cleaning properties of F_3_NO. Nonetheless, the limitation of this study is that only NF_3_ and F_3_NO plasmas were compared. In addition, EDS measurements were performed in parallel to assess the possibility of chemical contamination of the surface by ions in F3NO and phenomena occurring on the SiO_2_ surface during etching. As a result of the measurement, no chemical contamination was observed during etching with NF_3_ plasma or F_3_NO. Unlike NF_3_ plasma etching, it was observed that the mass ratio of oxygen of the SiO_2_ surface decreased during F_3_NO plasma etching. This may be attributed to the de-oxidation process of the SiO_2_ surface by NO ions.

The characteristics of the F_3_NO plasma were identified through these results, and the potential for replacing F_3_NO with NF_3_ was confirmed. Further studies will be needed when inert gases, such as Ar or He, are used as diluent. In addition, higher pressures need to be evaluated for the cleaning ability.

## Data Availability

Data available on reasonable request.
